# ﻿Revision of the genus *Scopoides* Platnick, 1989 from China, with description of a new genus (Araneae, Gnaphosidae)

**DOI:** 10.3897/zookeys.1172.105034

**Published:** 2023-07-26

**Authors:** Bo Liu, Feng Zhang

**Affiliations:** 1 The Key Laboratory of Zoological Systematics and Application, Institute of Life Science and Green Development, College of Life Sciences, Hebei University, Baoding, Hebei 071002, China Hebei University Baoding China

**Keywords:** Ground spider, morphology, new combination, new species, taxonomy

## Abstract

The genus *Scopoides* Platnick, 1989 from China is revised. A new genus, *Platnickus***gen. nov.**, is established with the type species *Scopoidesxizangensis* Hu, 2001, and a new species is described, *Platnickusreni***sp. nov.** Three new combinations are proposed: *Allozelotesgyirongensis* (Hu, 2001), **comb. nov.**, *Platnickuswanglangensis* (Yuan, Zhao & Zhang, 2019), **comb. nov.**, and *Platnickusxizangensis* (Hu, 2001), **comb. nov.**

## ﻿Introduction

*Scopoides* Platnick, 1989 is a small genus of Gnaphosidae, with 12 named species occurring in the USA and Mexico, and three species in China (WSC 2023). *Scopoides* was first reported from China (Xizang) by Hu (2001), who described two new species, *S.gyirongensis* Hu, 2001 and *S.xizangensis* Hu, 2001. Yuan et al. (2019) reported a third species from China (Sichuan), *S.wanglangensis* Yuan, Zhao & Zhang, 2019. *Scopoides* in North America was revised by Platnick and Shadab (1976), but species from China have never been revised and could be misplaced.

While examining *Scopoides* specimens from southwestern China, we compared them with generotype described in Platnick and Shadab (1976) and found none of them belong to *Scopoides*. The goals of this paper are to revise *Scopoides* in China, including a redescription of the type specimens of *S.gyirongensis* and *S.xizangensis*, propose three new combinations, and describe a new genus and a new species.

## ﻿Material and method

All specimens preserved in 75% ethanol were examined and measured under a Leica M205A stereomicroscope. The photographs of the genitalia and chelicerae were taken using an Olympus BX51 microscope or a Leica DM6000 B microscope equipped with a Kuy Nice CCD camera and were imported into Helicon Focus v. 7 for stacking. Bodies were photographed using a Leica M205A stereomicroscope. Final figures were retouched in Adobe Photoshop 2020. All measurements are given in millimeters. Leg measurements are shown as: total length (femur, patella, tibia, metatarsus, tarsus). Vulvae were cleared with Pancreatin (BBI Life Sciences). All specimens studied are deposited in the Museum of Hebei University (**MHBU**), Baoding, China.

Abbreviations used: **AG**, accessory gland; **AER**, anterior eye row; **AH**, anterior hoods; **ALE**, anterior lateral eyes; **AME**, anterior median eyes; **BG**, Bennett’s gland; **BH**, basal hematodocha; **C**, conductor; **CD**, copulatory duct; **CO**, copulatory opening; **d**, dorsal; **E**, embolus; **EB**, embolar base; **ED**, embolar denticle; **EP**, embolar base process; **ET**, epigynal teeth; **FD**, fertilization ducts; **Fe**, femur; **H**, hoods; **MA**, median apophysis; **MaAm**, major ampullate gland spigots; **MP**, mating plug; **Mt**, metatarsus; **p**, prolateral; **Pa**, patella; **PER**, posterior eye row; **PET**, petioles; **Pi**, piriform gland spigots; **PLE**, posterior lateral eyes; **PME**, posterior median eyes; **PO**, pocket; **PS**, primary spermathecae; **r**, retrolateral; **R**, radix; **SD**, sperm duct; **SE**, septum; **SS**, secondary spermathecae; **ST**, subtegulum; **T**, tegulum; **Ta**, tarsus; **TA**, tibial apophysis; **Ti**, tibia; **v**, ventral.

## ﻿Taxonomy

### ﻿Family Gnaphosidae, Banks, 1892

#### ﻿Genus Allozelotes

Taxon classificationAnimaliaAraneaeGnaphosidae

Yin & Peng, 1998

AE5DAF1C-BDF1-5DD5-82AF-1B937866877C

##### Type species.

*Allozeloteslushan* Yin & Peng, 1998.

##### Diagnosis.

See Yin and Peng (1998).

##### Comments.

This genus includes only four species from China, two of them (*A.microsaccatus* Yang, Zhang, Zhang & Kim, 2009 and *A.songi* Yang, Zhang, Zhang & Kim, 2009) are known only from females (WSC 2023).

##### Distribution.

China (Hunan, Jiangxi, Yunnan).

#### ﻿ Allozelotes gyirongensis

Taxon classificationAnimaliaAraneaeGnaphosidae

(Hu, 2001) comb. nov.

695C1F1D-04CB-5E95-805E-8969A2CF50D4

Figs 1 , 2 (吉隆异狂蛛)

Scopoides gyirongensis Hu, 2001: 267, fig. 152 (♀).
Scopoides gyirongensis : Song et al. 2004: 206, fig. 122 (♀).

##### Type material.

***Holotype*** ♀, China: Xizang Autonomous Region, Gyirong County, 2800 m elev., 17.XII.1983, Y. Yan leg., examined.

##### Diagnosis.

This species resembles *A.microsaccatus* in having a similar epigyne structure, but it can be distinguished by the medially procurved and weakly sclerotized anterior folds (vs anterior folds relatively straight and strongly sclerotized medially) (Fig. 2).

##### Redescription.

**Figure 1.**
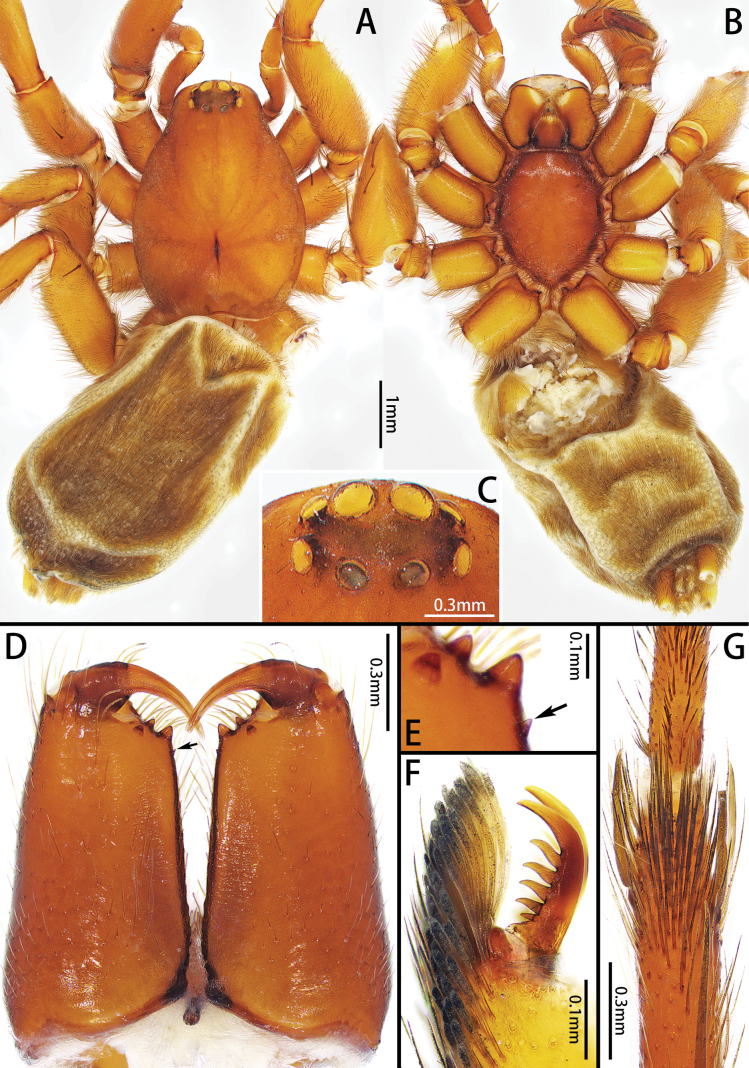
*Allozelotesgyirongensis* comb. nov., female **A, B** habitus, dorsal and ventral view **C** eye region, dorsal view **D** chelicerae, ventral view **E** details of chelicera, ventral view **F** tarsus IV, claw and claw tuft, lateral view **G** metatarsus IV, preening brush, ventral view.

**Female.** Holotype: total length 8.21; carapace 3.30 long, 2.65 wide; abdomen 4.90 long, 3.01 wide. Eye sizes and interdistances: AME 0.20, ALE 0.14, PME 0.15, PLE 0.17, AME–AME 0.05, AME–ALE 0.02, PME–PME 0.13, PME–PLE 0.08, ALE–PLE 0.03; posterior eye row procurved. Leg measurements: I 8.64 (2.66, 1.39, 2.03, 1.45, 1.11), II 7.81 (2.40, 1.45, 1.65, 1.30, 1.01), III 7.30 (2.00, 1.02, 1.78, 1.50, 1.00), IV 10.60 (2.90, 1.31, 2.49, 2.90, 1.00). Leg spination: I: Fe d2 p1, Ti v2, Mt v1; II: Fe d4 p1 r1, Ti v2, Mt v2; III: Fe d2 p2, Pa p1 r1, Ti d1 p2 r2 v3, Mt d2 p3 r2 v6; IV: Fe d2 p1, Pa r1, Ti p3 r3 v6, Mt d1 p3 r1 v6. Cheliceral promargin with 3 teeth, retromargin with 1 tooth (Fig. 1D, E). Color in alcohol (Fig. 1A, B): carapace yellow-brown; cephalic groove and radial furrow black; fovea distinct, longitudinal. Legs yellow-brown. Abdomen grey, without markings.

**Figure 2.**
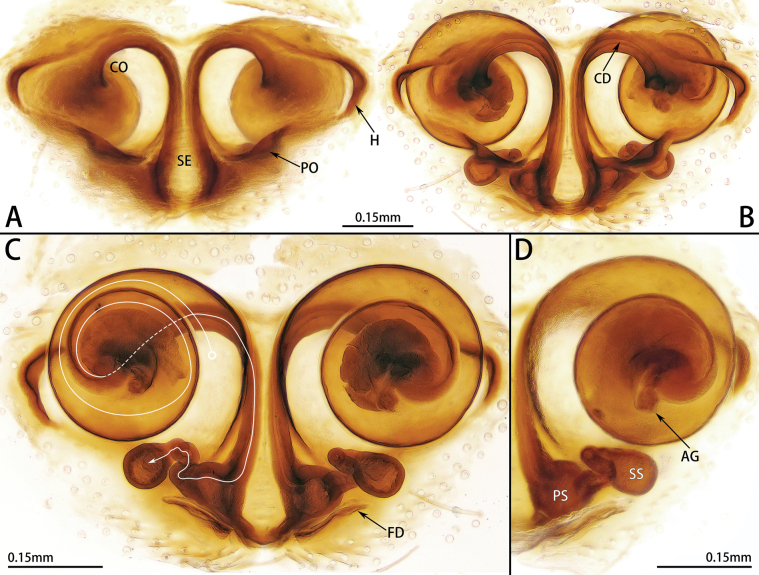
Female genitalia of *Allozelotesgyirongensis* comb. nov. **A, B** ventral view **C, D** dorsal view.

***Epigyne*** (Fig. 2). Epigynal plate diamond-shaped. Atrium almost two times wider than long. Septum wide posteriorly, stem almost two times thinner than base. Anterior folds procurved and weakly sclerotized medially, forming hoods posteriorly. Lateral folds form pockets. Copulatory opening large, located anteriorly. Copulatory ducts long, coiled, sclerotized proximally, and membranous distally. Accessory glands fist-shaped. Primary spermathecae small and globular, located at posterolateral part of septum, separated by their own diameter. Secondary spermathecae oval, almost equal to primary spermathecae in size. Fertilization ducts slender, laterally directed.

**Male.** Unknown.

##### Distribution.

China (Xizang).

##### Remarks.

The presence of long, snail-shell-shaped, convoluted copulatory ducts, fist-shaped accessory glands, and the secondary spermathecae almost the same size as the primary spermathecae indicate that this species is a member of *Allozelotes*, rather than *Scopoides*.

Yin and Peng (1998) placed *Allozelotes* as a zelotine spider based on the presence of a preening comb on metatarsi III and IV. Azevedo et al. (2017) placed this genus in Zelotinae based on Yin and Peng’s work. However, while examining the type specimens of *A.gyirongensis* comb. nov., we found a preening brush rather than preening comb on metatarsi III and IV (Fig. 1G) which indicates that *Allozelotes* is not a member of Zelotinae. *Allozelotes* is not assigned to any of the known subfamilies of Gnaphosidae (Azevedo et al. 2017; Lin and Li 2020), and it is unplaced here.

#### ﻿Genus Platnickus gen. nov.

Taxon classificationAnimaliaAraneaeGnaphosidae

64D69BB4-3A5C-58C9-866C-5E6101097149

https://zoobank.org/65D2E231-A79F-496E-9F47-743EB0160094

##### Type species.

*Scopoidesxizangensis* Hu, 2001.

##### Etymology.

The genus name is in honor of Norman I. Platnick (1951–2020) and his extensive contributions to arachnology; masculine in gender.

##### Diagnosis.

*Platnickus* gen. nov. resembles *Allozelotes* by the presence of a procurved PER, the presence of one retromarginal tooth, and the presence of a preening brush on metatarsi III and IV, but it can be distinguished by: 1) the slightly curved embolus, directed clockwise (vs embolus strongly curved, directed counterclockwise); 2) the absence of a macrosetae on the tibial apophysis; 3) the short copulatory ducts (vs long, convoluted copulatory ducts); and 4) the presence of long ducts leading to the secondary spermathecae and the secondary spermathecae with many glands.

*Platnickus* gen. nov. differs from *Scopoides* by the: 1) presence of a preening brush on metatarsi III and IV; 2) presence of a radix; 3) medially originating embolus (vs basally originating); 4) absence of ventral and dorsal points on the tibial apophysis; 5) copulatory opening located posteriorly (vs located anteriorly); and 6) presence of long ducts leading to the secondary spermathecae and the secondary spermathecae with many glands.

##### Description.

**Figure 3.**
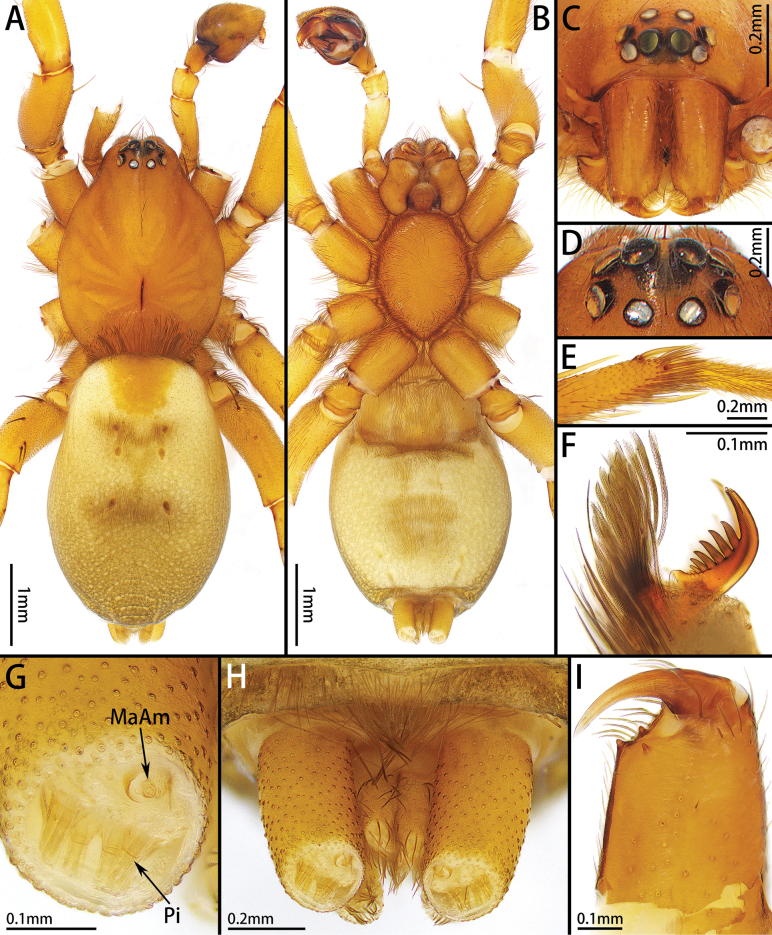
*Platnickusxizangensis* comb. nov., male **A–C** habitus, dorsal, ventral, and frontal view **D** eye region, dorsal view **E** metatarsus IV, preening brush, ventral view **F** tarsus IV, claw and claw tuft, lateral view **G** spigots on anterior lateral spinneret **H** spinnerets, ventral view **I** chelicerae, ventral view.

Medium-sized (total length: males = 5.63–7.61; females = 6.32–7.01). Carapace smooth, elongate-ovoid in dorsal view, highest at eye area, widest at coxae II and III. From above, AER straight; PER procurved; PME oblique, flat (Figs 3D, 5C, 7C). Cheliceral promargin with 2 or 3 teeth, retromargin with 1 tooth (Figs 3I, 5E, 7D). Leg spination: I: Fe d2/3 p1/2 r0/1, Ti v2/4, Mt v0/2; II: Fe d2/3 p1/2 r0/1, Ti v3/4, Mt v1/2; III: Fe d2/3 p1/2 r2, Pa p0/1 r1, Ti p3/5 r3/4 v4/5, Mt d1/2 p3 r2/3 v4/6; IV: Fe d2/3 p0/2 r2/3, Pa r1, Ti p3/5 r3/5 v5/6, Mt d1 p3 r3 v5/6. Leg formula: 4123. Female palp with claw (Fig. 5D). Trochanters not notched. Metatarsi III and IV with preening brush (Fig. 3E). Claw on tarsi IV with 8 teeth and claw tuft well developed (Fig. 3F). Anterior lateral spinnerets slightly longer than others, with 5 or 6 enlarged piriform gland spigots (Fig. 3G, H). Color in alcohol (Figs 3A, B, 5A, B, 7A, B): carapace pale or yellow-brown; cephalic groove and radial furrow black; fovea distinct, longitudinal. Legs pale or yellow-brown. Abdomen light grey, with anterior dorsal scutum and 3 pairs of muscle impressions medially.

**Figure 4.**
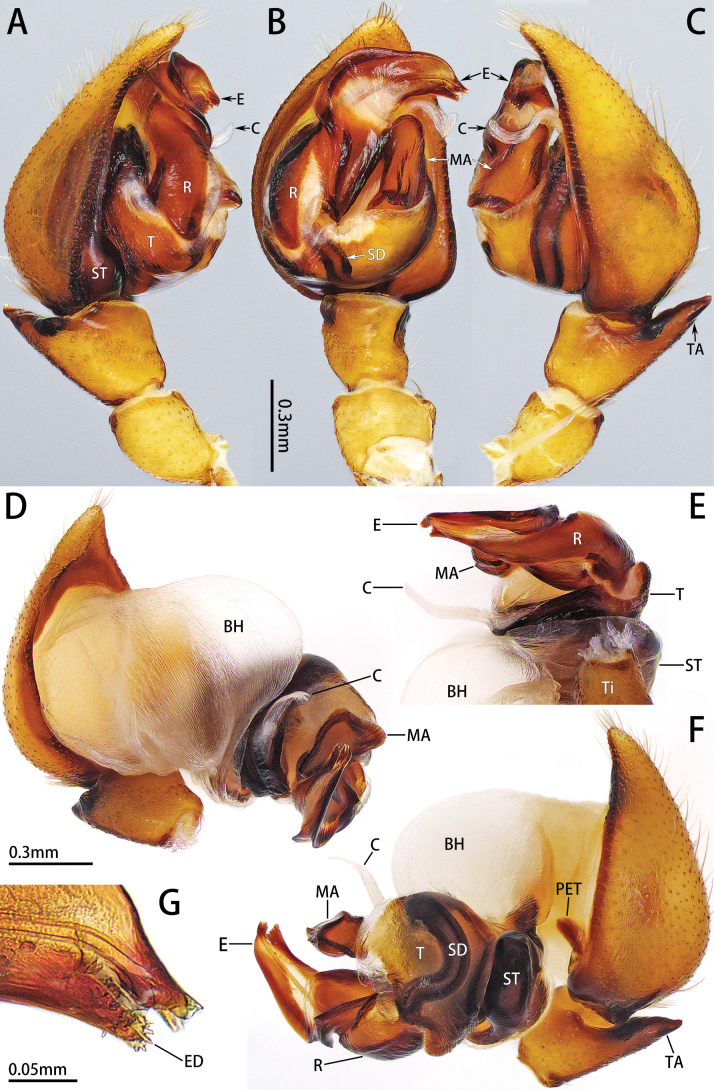
Palp of *Platnickusxizangensis* comb. nov., male **A, B** prolateral, ventral and retrolateral view **D–F** expanded palp, prolateral, posterior and retrolateral view **G** embolus tip.

***Male palp*** (Figs 4, 8): femur and patella unmodified. Cymbium pear-shaped, without claw. Basal hematodocha large, well developed. Tibial apophysis pointed, almost length of tibia, located dorsally. Tegulum larger than subtegulum. Conductor ribbon-shaped. Distal tubular membrane present. Median apophysis irregular polygon shaped. Radix elongate. Embolus originates at about 8–9 o’clock, with several ridges, bifurcated or notched distally.

**Figure 5.**
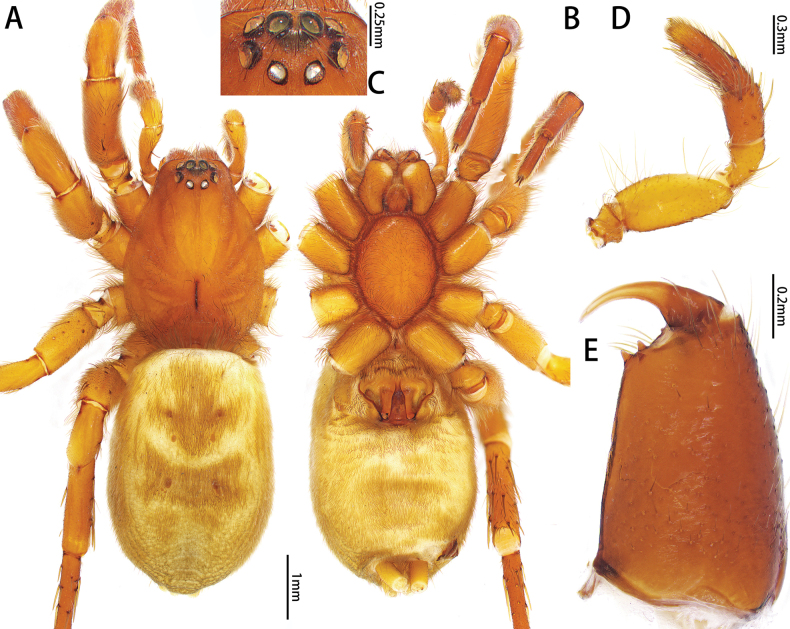
*Platnickusxizangensis* comb. nov., female **A, B** habitus, dorsal and ventral view **C** eye region, dorsal view **D** left palp, retrolateral view **E** chelicera, ventral view.

***Epigyne*.** See below (description in *P.xizangensis*).

##### Remarks.

Currently *Platnickus* gen. nov. is not assigned to any of the known subfamilies of Gnaphosidae (Azevedo et al. 2017; Lin and Li 2020); it is unplaced.

##### Distribution.

China (Xizang, Sichuan).

##### Composition.

*Platnickusxizangensis* (Hu, 2001), *P.reni* sp. nov., *P.wanglangensis* (Yuan, Zhao & Zhang, 2019).

#### ﻿ Platnickus xizangensis

Taxon classificationAnimaliaAraneaeGnaphosidae

(Hu, 2001) comb. nov.

0D218924-491E-5ED6-BCF4-3F5B798E7C25

**Figure 6.**
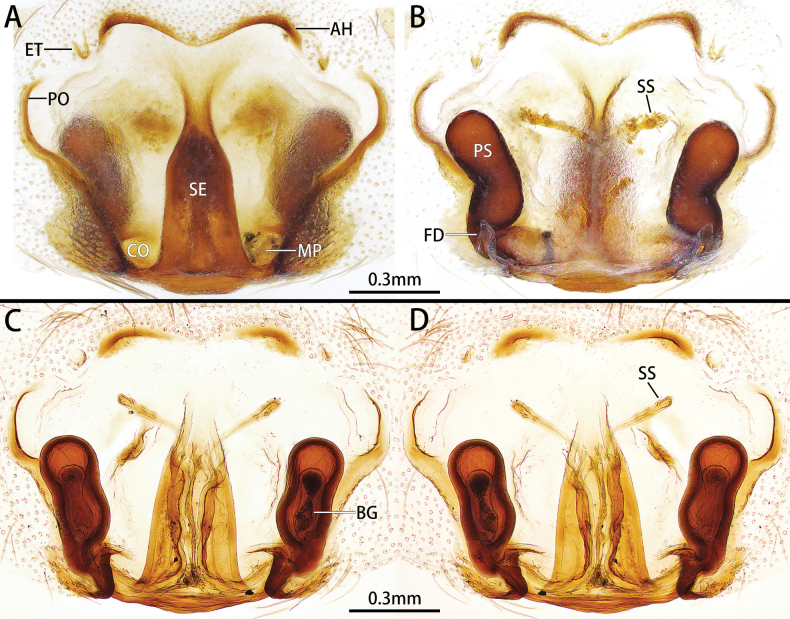
Female genitalia of *Platnickusxizangensis* comb. nov. **A, C** ventral view **B, D** dorsal view.

Figs 3 , 4 , 5 , 6 (西藏普蛛)

Scopoides xizangensis Hu, 2001: 268, fig. 153 (♀♂).
Scopoides xizangensis : Song et al. 2004: 207, fig. 123 (♀♂).

##### Type material.

***Holotype***: ♀, China: Xizang Autonomous Region, Linzhi City, 3000 m elev., 10.VIII.1987, F. Zhang leg. ***Paratype***: 3♀ 2♂, same data as holotype, examined.

##### Other material examined.

1♂, China: Xizang Autonomous Region, Bome County, 14.VIII.2002, M. Zhu & F. Zhang leg.

##### Diagnosis.

Males resemble *P.wanglangensis* in having a similar palp, but they can be distinguished by the presence of a curved embolus (vs embolus relatively straight) and the relatively flat median apophysis (vs median apophysis somewhat twisted) (Fig. 4).

##### Redescription.

**Male.** Total length 5.63–6.79. One paratype: total length 6.16; carapace 2.58 long, 2.08 wide; abdomen 3.57 long, 2.35 wide. Eye sizes and interdistances: AME 0.18, ALE 0.16, PME 0.13, PLE 0.18, AME–AME 0.05, AME–ALE 0.02, PME–PME 0.10, PME–PLE 0.05, ALE–PLE 0.04. Leg measurements: I 6.52 (1.93, 1.02, 1.55, 1.12, 0.90), II 6.26 (1.80, 0.97, 1.52, 1.09, 0.88), III 5.57 (1.58, 0.76, 1.23, 1.19, 0.81), IV 7.61 (2.07, 1.06, 1.75, 1.79, 0.94). Leg spination: I: Fe d3 p2 r1, Ti v4, Mt v2; II: Fe d3 p2 r1, Ti v4, Mt v2; III: Fe d3 p2 r2, Pa p1 r1, Ti p5 r4 v5, Mt d2 p3 r2 v6; IV: Fe d3 p2 r2, Pa r1, Ti p5 r5 v6, Mt d1 p3 r3 v6. Cheliceral promargin with 2 teeth. Color in alcohol (Fig. 3A, B): carapace and legs light brown.

***Palp*** (Fig. 4). Conductor ribbon-shaped. Median apophysis relatively flat and long, almost 1/2 length of bulb. Embolus broad, width/length ratio nearly 1/2, enwrapped by elongated radix. Embolus tip bifurcated, with many embolar denticles posteriorly.

**Female.** Total length 6.32–7.01. Holotype: total length 6.75; carapace 2.89 long, 2.21 wide; abdomen 3.81 long, 2.58 wide. Eye sizes and interdistances: AME 0.19, ALE 0.17, PME 0.16, PLE 0.18, AME–AME 0.04, AME–ALE 0.02, PME–PME 0.10, PME–PLE 0.07, ALE–PLE 0.02. Leg measurements: I 6.03 (1.78, 0.90, 1.51, 1.03, 0.81), II 5.57 (1.69, 0.91, 1.37, 0.84, 0.76), III 5.19 (1.50, 0.75, 1.06, 1.15, 0.73), IV 7.38 (2.01, 1.00, 1.74, 1.75, 0.88). Leg spination: I: Fe d3 p2, Ti v3, Mt v2; II: Fe d3 p2 r1, Ti v3, Mt v2; III: Fe d3 p2 r2, Pa p1 r1, Ti p4 r4 v5, Mt d2 p3 r2 v6; IV: Fe d3 p2 r2, Pa r1, Ti p4 r5 v6, Mt d1 p3 r3 v6. Chelicerae (Fig. 5E) as in male. Color in alcohol (Fig. 5A, B) as in male.

***Epigyne*** (Fig. 6). Epigynal plate irregularly hexagon-shaped, width/length ratio almost 7/5, with large atrium. Anterior folds M-shaped, forming hoods posteriorly. Lateral folds form pockets antero-laterally. Septum wide posteriorly, stem almost 3 times thinner than base. Copulatory opening large, distinct, located posteriorly. Copulatory ducts well sclerotized and short. Primary spermathecae large, peanut-shell-shaped. Secondary spermathecae with long ducts and many glands. Bennett’s gland long, oval, with constriction medially. Fertilization ducts laterally directed.

##### Distribution.

China (Xizang).

#### ﻿ Platnickus reni sp. nov.

Taxon classificationAnimaliaAraneaeGnaphosidae

777CE7E3-0C84-583D-ABC2-6170509A713A

https://zoobank.org/80C1C20A-464C-4116-BC49-89689FDFEFEF

Figs 7 , 8 (任氏普蛛)

##### Type material.

***Holotype***: ♂, China: Sichuan Prov., Barkam City, Mount Zhangqia, 3100 m elev., 27.VII.1999, G. Ren leg.

##### Etymology.

The species is named after the collector Prof. Guodong Ren (Hebei University, Baoding, China).

##### Diagnosis.

The male resembles *P.xizangensis* in having a similar palp, but it can be distinguished by the presence of embolar base process (EP1 and EP2), the notched embolus tip (vs bifid), the spoon-shaped conductor in retrolateral view (vs ribbon shaped), and the relatively short median apophysis, almost 1/3 length of bulb (vs relatively long, almost 1/2 length of bulb) (Figs 4, 8).

##### Description.

**Figure 7.**
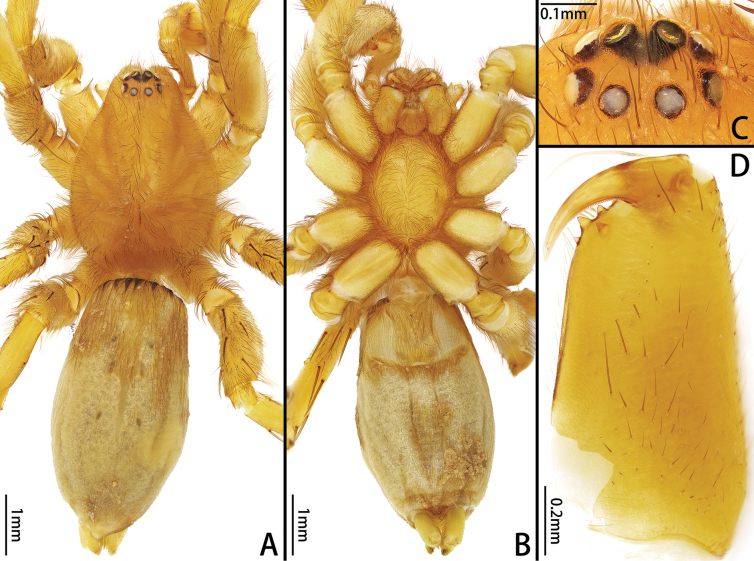
*Platnickusreni* sp. nov., male **A, B** habitus, dorsal and ventral view **C** eye region, dorsal view **D** chelicera, ventral view.

**Male.** total length 5.71; carapace 3.38 long, 2.33 wide; abdomen 4.13 long, 2.27 wide. Eye sizes and interdistances: AME 0.18, ALE 0.17, PME 0.14, PLE 0.19, AME–AME 0.08, AME–ALE 0.03, PME–PME 0.10, PME–PLE 0.08, ALE–PLE 0.05. Leg measurements: I 8.86 (2.57, 1.33, 2.17, 1.59, 1.20), II 8.15 (2.37, 1.28, 1.94, 1.47, 1.09), III 7.39 (2.11, 1.05, 1.62, 1.60, 1.01), IV 10.62 (2.89, 1.39, 2.34, 2.90, 1.10). Leg spination: I: Fe d2 p1, Ti v2; II: Fe d2 p1, Ti v3, Mt v1; III: Fe d2 p1 r2, Pa r1, Ti p3 r3 v4, Mt d1 p3 r3 v4; IV: Fe d2 r3, Pa r1, Ti p3 r3 v6, Mt d1 p3 r3 v6. Cheliceral promargin with 3 teeth (Fig. 7D). Color in alcohol (Fig. 7A, B): carapace and legs yellow-brown.

**Figure 8.**
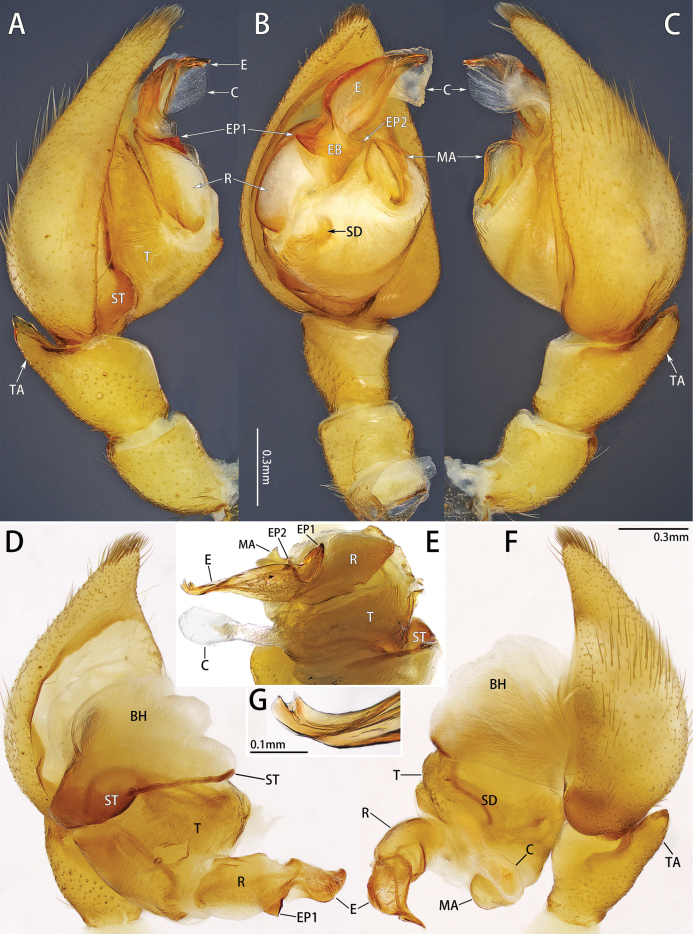
Palp of *Platnickusreni* sp. nov., male **A, B** prolateral, ventral and retrolateral view **D–F** expanded palp (BH is broken), prolateral, anterior and retrolateral view **G** embolus tip.

***Palp*** (Fig. 8). Conductor spoon-shaped in retrolateral view. Median apophysis relatively short, almost 1/3 length of bulb. Subtegulum slender anteriorly. Embolus twisted distally, with several ridges that form processes (EP1 and EP2). Embolus tip notched.

**Female.** Unknown.

##### Distribution.

China (Sichuan).

#### ﻿ Platnickus wanglangensis

Taxon classificationAnimaliaAraneaeGnaphosidae

(Yuan, Zhao & Zhang, 2019) comb. nov.

3E3CF867-8C9F-513A-8E05-66F2E037473B

Scopoides wanglangensis Yuan, Zhao & Zhang, 2019: 22, fig. 16 (♂).

##### Type material.

***Holotype***: ♂, China: Sichuan Province, Pingwu County, Wanglang Nature Reserve, Zhugencha, Baisha Valley, 32°52.729′N, 104°02.779′E, 2948 m elev., 15.V.2018, Z, Zhang et al. leg., not examined.

##### Diagnosis.

Males resemble *P.xizangensis* in having a similar palp, but they can be distinguished by the presence of a relatively straight embolus (vs relatively curved embolus) and the relatively twisted median apophysis (vs median apophysis relatively flat) (Fig. 4).

##### Description.

**Male.** See Yuan et al. (2019).

##### Remarks.

The illustrations and descriptions by Yuan et al. (2019: 22, fig. 16) show the same characteristics as in *Platnickus* gen. nov. For example, 1) the procurved posterior eye row; 2) the presence of three promarginal teeth and one retromarginal cheliceral tooth; 3) the presence of a radix; 4) the medially originating embolus with several ridges; 5) the irregular polygon-shaped median apophysis; and 6) the dorsally located tibial apophysis. These characters indicate that this species should be placed in *Platnickus* gen. nov.

##### Distribution.

China (Sichuan).

## Supplementary Material

XML Treatment for Allozelotes

XML Treatment for Allozelotes gyirongensis

XML Treatment for Platnickus

XML Treatment for Platnickus xizangensis

XML Treatment for Platnickus reni

XML Treatment for Platnickus wanglangensis

## ﻿Additional information

### Author ORCIDs

Bo Liu https://orcid.org/0000-0003-3740-7173

Feng Zhang https://orcid.org/0000-0002-3347-1031

### Data availability

All of the data that support the findings of this study are available in the main text.
